# Medical Classes in the West for 1834

**Published:** 1833

**Authors:** 


### Art. XIX. Medical Classes in the West for 1834.

*Medical College of Ohio*, 113, of whom 10 are beneficiaries.
*Transylvania University*, in Lexington, Ky., 260.
Dr. McDowell's Anatomical Class, same place, 110.

